# Ononin promotes survival rate in cecal ligation puncture-induced sepsis rat model by regulating inflammatory pathway

**DOI:** 10.1590/acb407825

**Published:** 2025-11-10

**Authors:** Bo Liu, Jing Wang, Ying Duan, Shaik Althaf Hussain, Alok Shiomurti Tripathi, Kang Li

**Affiliations:** 1 Xianyang Central Hospital – Department of Critical Care Medicine – Xianyang – China.; 2 Shaanxi University of Traditional Chinese Medicine – The Second Affiliated Hospital – Department of Obstetrics – Xianyang – China.; 3 King Saud University – College of Science – Department of Zoology – Riyadh – Saudi Arabia.; 4 ERA University – College of Pharmacy – Lucknow – India.

**Keywords:** Sepsis, Cytokines, Inflammation, Toll-Like Receptor 2, Interleukins

## Abstract

**Purpose:**

To investigate the potential protective effects of ononin on cecal ligation puncture (CLP) induced sepsis rat model.

**Methods:**

CLP was used to induce sepsis in rats and then treated with ononin at doses of 25 and 50 mg/kg intraperitoneally for seven days. The daily assessment included measurements of food and water intake, as well as body weight in the different rat groups. The study also examined the effects of ononin on survival rates, level of tumor necrosis factor (TNF)-α, interleukin (IL)-16, IL-6, C-reactive protein in the serum, and markers of oxidative stress in tissue homogenates of rats. The weight of spleen and lung tissue were also estimated in ononin-treated sepsis group. Additionally, histopathological examinations of lung and liver tissues were conducted in sepsis rats using hematoxylin and eosin staining.

**Results:**

The ononin-treated group showed significant improvements in food and water intake, as well as body weight, compared to the sepsis group of rats. Survival rate was also improved in the ononin-treated group. Ononin ameliorates oxidative stress and inflammatory mediators in sepsis rats. Histopathological changes (liver and lung tissue) were observed to be ameliorated in the group of rats treated with ononin *versus* the sepsis group.

**Conclusion:**

Ononin enhances survival rate in sepsis in rats by reducing inflammation and oxidative stress.

## Introduction

Sepsis is a systemic infection, causes an inflammatory response and has a mortality of up to 60%. It is the major cause of death among the patients hospitalized in the intensive critical care unit^1^. Moreover, the advancement of the medical field in recent times has not been able to reduce incidences of mortality even in developed countries. Cytokines level outburst and macrophage activation occur due to bacterial endotoxin^2^. Bacterial lipopolysaccharide enhances the production of reactive oxygen species (ROS), which activates inflammation process and alters the cellular process, leading to cell injury^3^. All these changes alter the physiological function of the organs and contribute to multiple organ dysfunction, commonly leading to sepsis and, also, mortality^4^. Advancement of medical sciences has improved pharmacotherapy in general. However, data suggest that there is no significant improvement observed in mortality rate among patients suffering from sepsis^5^. Thus, it is necessary to develop effective therapy to manage it.

In recent times, bioactive molecules from herbal origin have shown promising effects in sepsis management. Ononin is chemically an isoflavone isolated from *Millettia nitida, Pueraria lobata, Smilax scobinicaulis*, and *Astragalus membranaceus*, traditionally used as medicine in China^6^. It shows strong anti-inflammatory, antioxidant, and antiviral properties^7^^–^ 9. It protects alleviation of cartilage damage by reducing cytokine and NF-κB in rheumatoid arthritis^10^. Moreover, ononin shows neuroprotective effects against Alzheimer’s disease by reducing neuronal inflammation and oxidative stress, diminishing cytokine level^11^. It also reported to regulate cellular apoptosis and proliferation like biological properties, which contribute to the management of neuronal injury and obesity^12^.

The given investigation assessed the beneficial effects of ononin against sepsis.

## Methods

### Experimental protocol: in vivo

Healthy male Sprague-Dawley rats (175–200 g) were kept under standard conditions. Experimental study on animals was approved by institutional animal ethical committee (650/05/C/CPCSEA/2020/12). All the 30 rats were separated into five different groups:

- Sham group;
- CLP group;
- Standard (STD) group: it received 5 mg/kg of imipenem subcutaneously for seven days;
- Ononin 25 mg/kg: it received 25 mg/kg ononin intraperitoneally for seven days after induction of sepsis;
- Ononin 50 mg/kg: it received 50 mg/kg ononin intraperitoneally for seven days after induction of sepsis;

The cecal ligation puncture (CLP) method was used for sepsis induction. The animals were briefly anesthetized, and laparotomy was performed to expose the intestine in aseptic conditions^13^. The ileocecal junction was used to identify the cecum and puncture it with a needle to perforate the tissue after ligation with a 3.0-size silk suture. Thereafter, the incision was sutured with 4.0 size suture, and rats were separated individually in cage. Twelve hours later, the saline was provided. The perforation and ligation process was not performed on the sham group. The protective effect of ononin against sepsis was assessed by determining daily water and food intake and the survival rate of rats.

### Estimation of biochemical parameters

Inflammatory parameters such as C-reactive protein and cytokines—tumor necrosis factor (TNF)-α, interleukin (IL)-16, IL-6—were estimated in the serum of sepsis rats with the enzyme-linked immunosorbent assay (ELISA) method. Moreover, a liver function test was performed according to the method given in the kits.

### Estimation of the weight of different organs

Animals were sacrificed, and organs like the spleen and the lung were isolated from each animal. These organs were weighed individually with an electronic balance.

### Estimation of parameters of oxidative stress

Parameters of oxidative stress—malondialdehyde (MDA), superoxide dismutase (SOD), and reduced glutathione (GSH)—were determined in lung tissue homogenate as reported in studies^14^^–^ 16. Tissue homogenate (0.1 mL) was mixed with phosphate buffer (pH 8; 2 mL) and DTNB (0.2%; 0.5 mL), and 15 min later, the mixture was scanned for absorbance at 412 nm to estimate GSH level. Lipid peroxidation level was estimated by determining the level of MDA in the liver tissue homogenate at 532 nm after treating it with thiobarbituric acid as previously cited in studies. SOD activity was estimated in the liver tissue homogenate by observing the inhibition ability of the enzyme to nitroblue tetrazolium (NBT), which is represented as U/mg protein.

### Real-time quantitative reverse transcription polymerase chain reaction

Lung tissue homogenate was used to determine the mRNA expression of TNF-α, NF-κB, and TLR-2 in sepsis rats. TRIzol kit was used to isolate the total RNA content in the lung tissue according to the directions given in the kit. Further, real-time quantitative reverse transcription polymerase chain reaction (qRT-PCR) was performed as per the Fast Start Universal SYBR Green Master from single strand cDNA synthesis, and expression of the gene was estimated by ABI 7300 real time PCR system (Table 1).

**Table 1:** Primer details.

Sr. No.	Primer	Forward sequence	Reverse sequence
1.	TLR-2	CTTCACTCAGGAGCAGCAAGCA	ACACCAGTGCTGTCCTGTGACA
2	NF-κB	TGAACCGAAACTCTGGCAGCTG	CATCAGCTTGCGAAAAGGAGCC
3	TNF-α	CTCTTCTGCCTGCTGCACTTTG	ATGGGCTACAGGCTTGTCACTC

TNF-α: tumor necrosis factor-α.

Source: Elaborated by the authors.

### Histopathology study

Liver and lung tissues were fixed with formalin (10%) after isolation. Tissues were dehydrated with different concentrations of ethanol and then embedded into liquid paraffin to prepare the wax cubes. Tissues were sectioned with a microtome and stained with hematoxylin and eosin (H&E) staining, and analysis of histopathological changes was observed with a trinocular microscope.

### Statistical studies

Results were shown as mean ± standard error of the mean (SEM), and biostatistics was performed by analysis of variance (ANOVA) followed by Dunnett’s test using GraphPad Prism software version 9.3.1 (GraphPad Software, Inc., Boston, MA, United States of America). Values were considered significant at *p* < 0.05.

## Results

### Ononin ameliorates body weight and food and water intake

**Figure 1:**
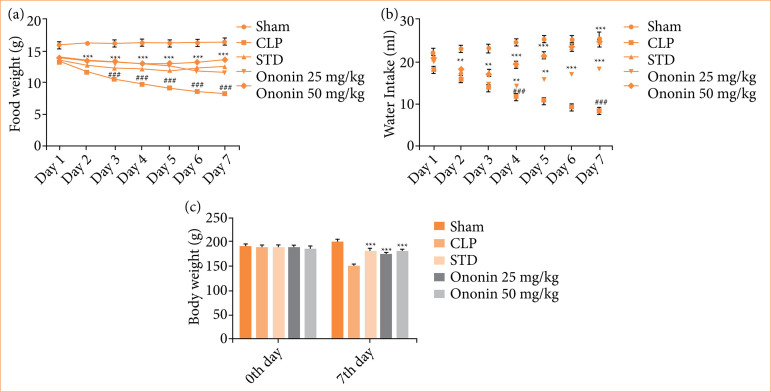
Effect of ononin on food and water intake and body weight in sepsis rats. **(a)** Assessment of daily food intake for seven days; **(b)** assessment of daily water intake for seven days; **(c)** assessment of body weight on the zero and the seventh day of protocol^!^.

CLP: cecal ligation puncture; STD: standard; ^!^mean ± standard error of the mean; ^##^ *p* < 0.01, and ^###^ *p* < 0.001 in comparison to sham group; **p < 0.01, and ***p < 0.001 in comparison to CLP group. Source: Elaborated by the authors.

The effect of ononin was estimated on the daily food and water intake and body weight of sepsis rats, as shown in Fig. 1. The CLP group showed a significant reduction in the water and food intake in comparison to the sham group, and treatment with ononin significantly (*p* < 0.01) improved it in sepsis rats (Figs. 1a and 1b). Body weight of the CLP group was reduced on the seventh day of protocol, which was reversed in ononin-treated sepsis rats (Fig. 1c).

### Ononin ameliorates the percentage of survival rate

**Figure 2:**
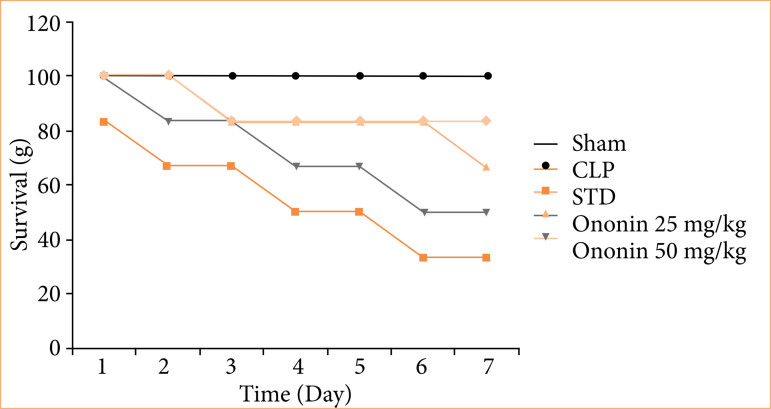
Effect of ononin on the percentage of survival rate in sepsis rats.

CLP: cecal ligation puncture; STD: standard. Source: Elaborated by the authors.

The percentage survival rate was observed among all the groups of rats, as shown in Fig. 2. The sham group showed 100% survival, which was reduced to 33.33% in the CLP group. Treatment with ononin enhanced the percentage of survival rate up to 66.66% in sepsis rats, as shown in Fig. 2.

### Ononin ameliorates liver function

**Figure 3:**
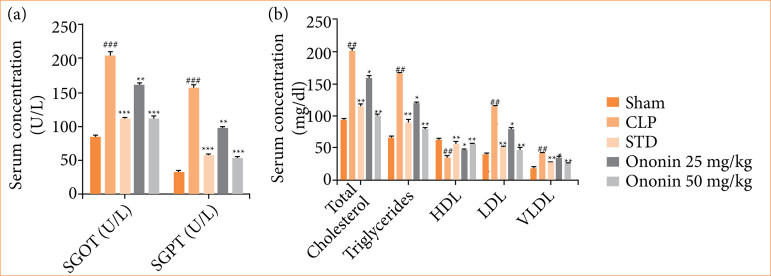
Effect of ononin on the liver function and lipid profile of sepsis rats^!^.

CLP: cecal ligation puncture; STD: standard; SGOT: serum glutamic-oxaloacetic transaminase; SGPT: serum glutamic pyruvic transaminase; HDL: high-density lipoprotein; LDL: low-density lipoprotein; VLDL: very low-density lipoprotein; !mean ± standard error of the mean; ^##^ *p* < 0.01, and ^###^ *p* < 0.001 in comparison to sham group; **p < 0.01, and ***p < 0.001 in comparison to CLP group. Source: Elaborated by the authors.

Liver function was assessed by determining the activity of serum glutamic-oxaloacetic transaminase (SGOT) and serum glutamic pyruvic transaminase (SGPT) in ononin-treated sepsis rats. The CLP group showed a significant (*p* < 0.001) increase in the level of SGOT and SGPT in comparison to the sham group. The level of these enzymes was reduced significantly (*p* < 0.001) in the serum of the ononin-treated group in comparison to the CLP group in a dose-dependent manner (Fig. 3a).

The CLP group showed a significant increase in lipid levels, such as total cholesterol, triglyceride, low-density lipoprotein (LDL), and very low-density lipoprotein (VLDL) and a decrease in high-density lipoprotein (HDL) level in comparison to the sham group of rats. Treatment with ononin ameliorated lipid profile among sepsis rats (Fig. 3b).

### Ononin ameliorates inflammatory cytokine

**Figure 4:**
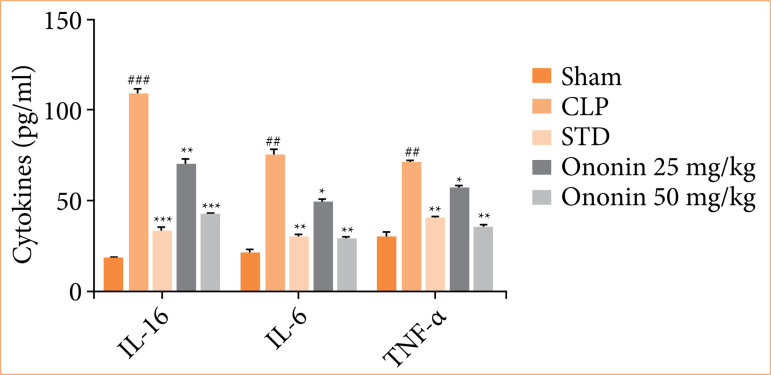
Effect of ononin on the level of inflammatory cytokines in the serum of sepsis rats^!^.

CLP: cecal ligation puncture; STD: standard; IL: interleukin; TNF: tumor necrosis factor; !mean ± standard error of the mean; ^###^ *p* < 0.001 in comparison to sham group; *p < 0.05, **p < 0.01, and ***p < 0.001 in comparison to CLP group. Source: Elaborated by the authors.

Inflammatory cytokines level was estimated in the serum of ononin-treated sepsis rats. The CLP group showed a significant (*p* < 0.001) increase in the levels of IL-16, IL-6, and TNF-α of CLP group in comparison to the sham group. All the levels were reduced in ononin-treated group of rats (Figure 4).

### Ononin ameliorates oxidative stress

**Figure 5:**
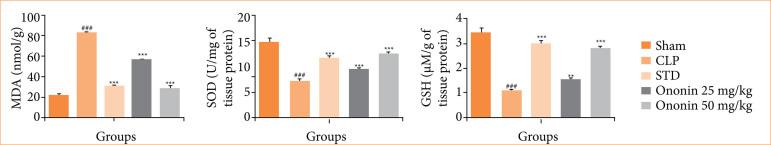
Effect of ononin on the oxidative stress markers such as MDA, SOD, and GSH in sepsis rats^!^.

CLP: cecal ligation puncture; STD: standard; MDA: malondialdehyde; SOD: superoxide dismutase; GSH: glutathione; !mean ± standard error of the mean; ^##^ *p* < 0.01, and ^###^ *p* < 0.001 in comparison to sham group; **p < 0.01, and ***p < 0.001 in comparison to CLP group. Source: Elaborated by the authors.

Oxidative stress markers such as MDA, SOD, and GSH were estimated in ononin-treated sepsis rats (Fig. 5). The CLP group showed a significant increase in the level of MDA and a reduction of GSH in comparison to the sham group. Moreover, activity of SOD reduced in CLP group compared to the sham group of rats. These altered parameters were ameliorated with ononin-treatment in sepsis rats.

### Ononin ameliorates organ weight

**Figure 6:**
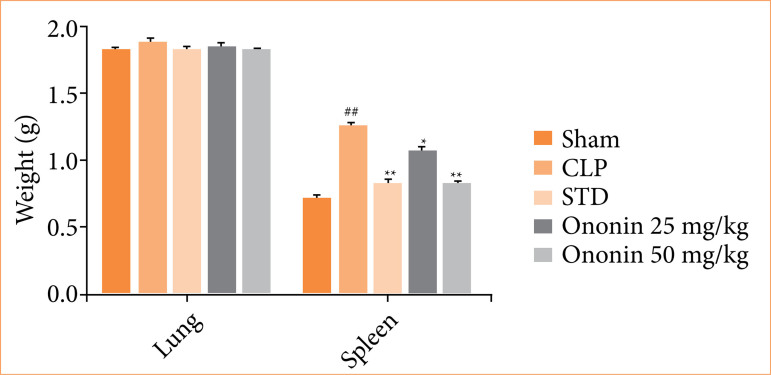
Effect of ononin on the lung and spleen weight of sepsis rats^!^.

CLP: cecal ligation puncture; STD: standard; !mean ± standard error of the mean; ^##^ *p* < 0.01, and ^###^ *p* < 0.001 in comparison to sham group; *p < 0.05, and **p < 0.01 in comparison to CLP group. Source: Elaborated by the authors.

Lung and spleen weight were estimated in ononin-treated sepsis rats, as shown in Fig. 6. There was no alteration in the weight of the lung among all the groups of rats. Spleen weight was enhanced significantly in the CLP group in comparison to the sham group of rats. However, ononin-treated group showed a significant reduction in the weight of sepsis rats.

### Ononin ameliorates mRNA expression of TNF-α, TLR-2, and NF-kB

**Figure 7:**
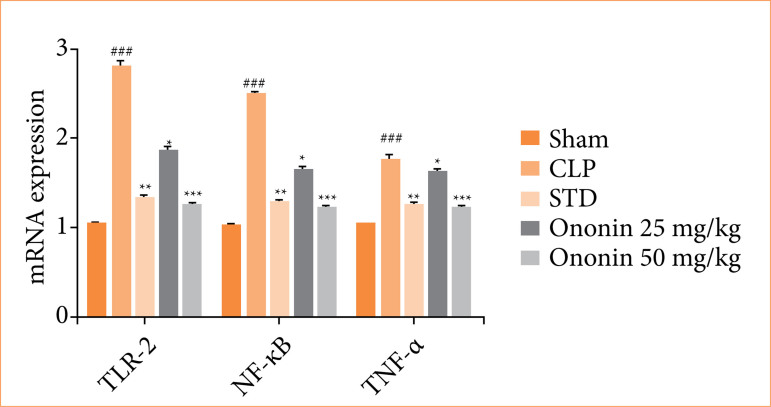
Effect of ononin on the relative mRNA expression of TLR-2, NF-kB, and TNF-α in the sepsis rats^!^.

CLP: cecal ligation puncture; STD: standard; TNF: tumor necrosis factor; !mean ± standard error of the mean; ^##^ *p* < 0.01, and ^###^ *p* < 0.001 in comparison to sham group; *p < 0.05, **p < 0.01, and ***p < 0.001 in comparison to CLP group. Source: Elaborated by the authors.

Relative mRNA expression of TLR-2, NF-kB, and TNF-α in the ononin-treated sepsis rats is presented in Fig. 7. There was a significant increase (*p* < 0.001) in relative mRNA expression of TLR-2, NF-kB, and TNF-α in the CLP group in comparison to the sham group of rats, and these expressions were reversed in ononin-treated groups.

### Ononin ameliorates histopathological changes in liver and lung tissue

**Figure 8:**
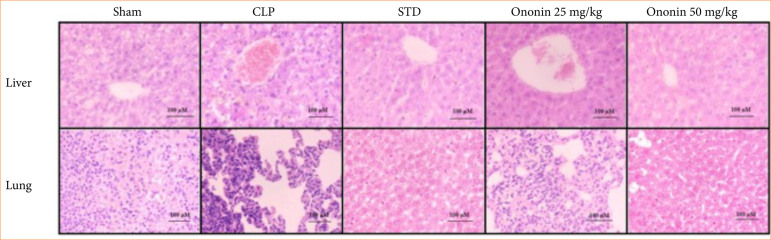
Assessment of the effect of ononin on histopathological changes in liver and lung tissue of CLP-induced sepsis rats by hematoxylin and eosin staining.

CLP: cecal ligation puncture; STD: standard. Source: Elaborated by the authors.

Histopathology changes were observed in lung and liver tissue in all the groups with H&E staining (Fig. 8). Lung tissue of the sham group was normal in appearance, and the sepsis group tissue was characterized by thick alveolar septa due to the filtration of inflammatory cells and severe congestion of blood vessels. The ononin- and STD-treated group showed reduced infiltration of inflammatory cells, and the septa appeared normal. Liver tissue also showed degenerative changes in hepatocytes and stenosis in the portal vein. However, ononin and the STD group showed a reduction in these degenerative changes in the liver tissue.

## Discussion

Sepsis is a systemic infection or inflammation and the cause of the majority of mortality cases hospitalized in the intensive critical care units. Sepsis management remains a challenge with the conventional drugs available for it. Thus, the present report determined the beneficial effect of ononin for sepsis management. CLP induced sepsis in an animal model, resembling the pathophysiology of sepsis as it with clinical conditions^17^. Moreover, induction of it with CLP has higher chances of development of disease in comparison to other models^18^. Our study induced sepsis with the CLP model and evaluated the effects of ononin against it.

Sepsis results due to infection and leads to patients’ hospitalization. It was observed that patients suffering from sepsis reduce food and water intake. This change causes a severe reduction in the body weight of patients suffering from sepsis^19^ — our report supports it. Effective sepsis management improves body weight among patients according to the literature^20^. Data of the study revealed that treatment with ononin improves the food and water intake and body weight of sepsis rats. Moreover, mortality is one of the major concerns with sepsis; as per the literature, the percentage of survival is very low in the CLP-induced sepsis model^21^, and our investigation also supports it. The result of the investigation suggests that the survival rate in the ononin-treated group significantly enhances in sepsis rats.

Sepsis causes multiple organ dysfunction, which leads to an increase in mortality. It majorly affects the function of visceral organs like the lung, heart, and liver^22^. SGOT and SGPT are the enzymes available in the liver and maintain its function. Moreover, the level of lipid profile is also regulated by the liver. The activity of these enzymes reduces, and the lipid profile is altered in patients suffering from liver dysfunction^23^. Literature suggests that liver function improves in patients recovered from sepsis^24^. There was a significant improvement in liver function in ononin-treated sepsis rats. Moreover, splenomegaly occurs in sepsis, as the spleen is the primary organ to regulate the function of immune cells, and systemic infection or inflammatory conditions provoke the proliferation of spleen cells^25^. Literature suggests that recovery of patients from sepsis reduces the splenomegaly condition^26^, and data of the given report showed a reduction in the weight of spleen among the ononin-treated group in comparison to the CLP group of rats.

Systemic infection alters the cellular function, which enhances the production of ROS and promotes the free radicals. These free radicals cause cellular injuries by damaging the cell membrane, lead to an increase in MDA level, as it is a content of the cell membrane. SOD scavenges superoxide anions, and the reduction of its activity generates free radicals and causes cell injury. GSH level is reduced in increased oxidative stress condition, which commonly occurs in sepsis condition^27^. These parameters of oxidative stress were altered in sepsis, and our investigation revealed that treatment with ononin attenuates the altered the level of oxidative stress parameters in sepsis rats.

TLR regulates immunomodulation inversely in cytokine storm associated with sepsis, as TLR-4 is present in human cells, which is activated in sepsis and promotes the expression of cytokines^28^. It was reported that NF-κB is activated with TLR-4 and promotes the level of TNF-α, IL-16, and IL-6 in the tissue of sepsis rats. These cytokines further enhance cell injury to different organs, leading to multiple organ dysfunction commonly observed in sepsis. Drugs used for sepsis management reduce cytokine storm, and the results of this study showed that treatment with ononin reduces the level of cytokines in sepsis rats.

## Conclusion

The investigation showed survival rate enhances with ononin treatment in CLP induced-sepsis rats. Ononin treatment ameliorated altered levels of oxidative stress and inflammatory cytokines in sepsis rats by promoting the TLR-4 pathway.

## Data Availability

All data generated or analyzed during this study are included in this article. Further enquiries can be directed to the corresponding author.
